# Predictions of children’s emotionality from evolutionary and epigenetic hypotheses

**DOI:** 10.1038/s41598-019-39513-7

**Published:** 2019-02-21

**Authors:** Jonathan Hill, Andrew Pickles, Nicola Wright, Elizabeth Braithwaite, Helen Sharp

**Affiliations:** 1 School of Psychology and Clinical Language Sciences, University of Reading, Reading, RG6 6AL UK; 2 Institute of Psychiatry, Psychology and Neuroscience, King College London, London, SE5 8AF UK; 3 Department of Psychological Science, Institute of Psychology, Health and Society, University of Liverpool, Liverpool, L69 3GB UK; 4 Department of Psychology, Manchester Metropolitan University, Manchester, M15 6BH UK

## Abstract

Sex-dependent effects of mismatched prenatal-postnatal maternal conditions are predicted by combining two evolutionary hypotheses: that foetal conditions provide a forecast of likely postnatal environments (Predictive Adaptive Response), and that the female foetus is better adapted than the male to maternal adversity (Trivers-Willard hypothesis). Animal studies have implicated glucocorticoid mechanisms modifiable by effects of postnatal tactile stimulation on glucocorticoid receptor gene expression. In this study we examined behavioural predictions in humans based on these evolutionary and epigenetic models. Mothers in a general population cohort provided self-reported anxiety scores at 20 weeks pregnancy, and at 9 weeks, 14 months and 3.5 years postpartum, and frequency of infant stroking at 9 weeks. Mothers and teachers reported child symptoms at 7 years. SEM models with maximum-likelihood estimates made use of data from 887 participants. There was a three-way interaction between prenatal and postnatal anxiety and maternal stroking in the prediction of irritability, seen only in girls. This arose because lower maternal stroking was associated with higher irritability, only in the mismatched, low-high and high-low maternal anxiety groups. We provide evidence that mechanisms likely to have evolved well before the emergence of humans, contribute to the development of children’s emotionality and risk for depression.

## Introduction

The question of whether evolutionary models can be brought to bear on the way questions in child development and psychiatry are formulated remains controversial. There are many reasons, including that when they are applied *post hoc*, they can explain almost anything, and that they are incapable of taking account of the complexity of social and linguistic processes found in humans, but not other species. However, some of the boldest and most productive hypotheses from the past 50 years have been generated by applying evolutionary considerations. Most notably, John Bowlby, bringing together evolutionary and psychoanalytic theory, argued that infants and young children seek out familiar caregivers in preference to other adults, not because of reinforcement which was the prevailing model in the 1960’s, but because of a biological predisposition to become attached^1,2^. As a result of human and non-human studies influenced by this proposal, Bowlby’s hypothesis is now non-controversial, and continues to be productive. Also working from an evolutionary perspective, Belsky theorised that there are selection advantages for children to vary in their susceptibility to both adverse and beneficial effects of rearing influences^3,4^. This led to the differential susceptibility hypothesis: that the children with the poorest behavioural outcomes in the context of harsh rearing environments will also have the best outcomes in supportive environments. This hypothesis has also received considerable empirical support^5^. A key consequence of the application of evolutionary considerations in these instances is that they have given rise to study designs and data analyses that would not have otherwise been considered. This is also the case for the analyses presented in this paper.

According to the Predictive Adaptive Response (PAR) hypothesis, cues received in early life influence the development of a phenotype that is adapted to the environmental conditions of later life. Such adaptations confer adaptive advantages when the predicted and actual environments are similar, but where they differ, the mismatch between the individual’s phenotype and the conditions in which it finds itself can have adverse consequences for Darwinian fitness and, later, for health^6,7^. There are numerous examples of this PAR mechanism across organisms as diverse as insects, crustacea and mammals^6,7^. The applicability of the PAR hypothesis in humans has been contested on a number of grounds, including that the mechanism would be unlikely to confer the proposed advantage in long lived species, notably humans, in which environmental change across generations is common^6,7^. However, well-replicated associations between low birth weight and diabetes, cardiovascular disease and obesity in later life, in industrialised societies indicate that the PAR mechanism, also referred to as ‘foetal programming’, may operate in humans over long periods of time^8^. It is hypothesised that low birth weight reflects an adaptive mechanism to survival in environments of food scarcity, with the risk for disease arising from the mismatch between the encountered high calorie environment and the anticipated environment of food scarcity. Within the fields of perinatal psychiatry and child development, the foetal programming and PAR hypotheses have been adapted to explain the association between exposure to maternal prenatal stress and offspring psychopathology. In this context, prenatal stress signals a hostile postnatal environment, whereby a rapidly distracted attention (attention deficit hyperactivity disorder; ADHD) and aggression may confer a survival advantage^9,10^, but simultaneously confers disadvantage in a non-threatening environment.

Foetal adaptations may however vary by sex of the foetus. According to the Trivers-Willard (T–W) hypothesis, if maternal health during pregnancy predicts later reproductive fitness in the offspring, then a male predominance of births will be favoured when maternal conditions are good, because healthy males compete successfully for females^11^. By contrast, when maternal conditions are poor the sex ratio will be reversed, both to avoid bearing males who compete less successfully for females, and also because, compared to females, health outcomes for mothers following male births are poorer^11^. Although this hypothesis has been subject to challenges and modifications^12^, the central idea that reproductive strategies associated with poor maternal conditions involve sacrifice of males and protection of females has received substantial support. It is consistent with well documented observations that male foetuses are more vulnerable to threats such as preterm birth, and are more likely to suffer neurodevelopmental consequences of foetal insults^13^. It also predicts better outcomes for females following poor maternal conditions, which is consistent with a body of literature that demonstrates more female placental adaptations in conditions of adversity^13^. However, if a protective effect in females arises from advantages conferred by foetal anticipation of matched environments (PAR hypothesis), mismatches between maternal conditions during pregnancy and the postnatal environment will create vulnerability. Combining the T-W and PAR hypotheses leads to the prediction that the effects of prenatal risks will operate differently in males and females. In females, vulnerability will be generated by particular combinations of prenatal and postnatal risks, but in males, poor outcomes will arise incrementally from degree of prenatal risk.

In support of these hypotheses, there is evidence from the human and animal literature that foetal programming mechanisms in the context of prenatal stress exposure are sex-specific, whereby a glucocorticoid-mediated mechanism increases risk for affective symptoms in females. For example, female, but not male, rats exhibit elevated anxiety and depression-like behaviours following exposure to *in utero* gestational stress^14^. The effect was eliminated by adrenalectomy of the pregnant dams^15^, consistent with risk mediated by glucocorticoid mechanisms that are specific to females. In the human literature, elevated maternal prenatal cortisol, the main glucocorticoid stress hormone, has been associated with the following outcomes in females, but not males: reduced foetal growth, an established risk factor for later depression^16^; infant negative emotionality^17,18^; fearful infant temperament^19^; affective problems in childhood^20^; pre-adolescent anxiety^19^, depression and a flattened diurnal cortisol profile in adolescence^21^.

The role of glucocorticoid mechanisms predicted by a combination of the PAR and T-W hypotheses, has been examined in starlings. Prenatal stress was mimicked by injection of corticosterone into starling eggs, and stressful postnatal conditions for chicks were created by wing clipping of mothers after hatching. Corticosterone levels in chicks following a standard stressor were greatest in those who had been exposed to the mismatch condition of no corticosterone injection followed by rearing by wing clipped mothers, and this effect was greater in females than males, as evidenced in a significant sex by matching condition interaction^22,23^. Furthermore, there was a higher mortality among male chicks from corticosterone injected eggs reared by wing clipped mothers, significantly shifting the sex ratio in favour of females.

Animal models also indicate that postnatal modification of prenatal effects can arise from maternal behaviours via glucocorticoid mechanisms. In rodents, high levels of maternal care during the postnatal period (indexed by frequent ‘licking and grooming’ behaviours (LG)) increases GR expression via promotor demethylation, which improves HPA regulation and reduces anxiety behaviours in offspring^24,25^. Thus, both prenatal stress and postnatal maternal caregiving behaviour act on the same biological system (HPA axis), and high levels of maternal postnatal care can reverse effects of prenatal stress. In animals, the postnatal effects are caused by tactile stimulation, leading us to hypothesise that similar effects might arise in humans from maternal stroking of infants. Based on self-reports at 5 and 9 weeks, we found that the frequency of maternal stroking modified associations between prenatal depression and infant temperament and vagal reactivity at age 29 weeks^26^, and between prenatal anxiety and child internalising symptoms at age 2.5 years^27^, and child internalising and externalising symptoms at age 3.5 years^28^. On each occasion the direction of effect of maternal stroking was the same; the effect of the prenatal risk on child outcomes was markedly reduced in the presence of high maternal stroking. At age 2.5 years, but not at age 3.5 years, the moderating effect of stroking on the association between prenatal anxiety and child internalising symptoms was significantly greater in girls than in boys. In this study we hypothesised that postnatal stroking will moderate the contributions of prenatal and postnatal anxiety because of our previous finding at age 2.5 years in relation to prenatal anxiety. However, foetal origins findings have also been reported for prenatal depression, so we also report analyses examining the role of maternal stroking in relation to prenatal and postnatal depression^29^.

These findings suggest that the mismatch effects that we predict for females on the basis of the PAR and T-W hypotheses, may also be modified by exposure to maternal stroking. We hypothesise that these mechanisms will contribute to depression in humans, for four main reasons. First, in animal studies, prenatal stress and low tactile stimulation are associated with depression-like behaviours mediated via altered HPA axis regulation^25^. Second, human sex-dependent associations between prenatal risks and depression, with the association seen only in females, have been shown in large longitudinal studies^30,31^. Third, studies have demonstrated a sex-dependent association between prenatal maternal cortisol levels and increased amygdala activation^32^, which is a well-established biomarker for depression^33^. Fourth, depression is associated with altered HPA axis regulation. While depression, an internalising disorder, commonly occurs for the first-time during adolescence, in girls it is predicted by childhood externalising symptoms of oppositional defiant disorder (ODD)^34^. Recent studies have clarified that there is probably a specific ‘irritability’ subset of ODD symptoms, characterized by touchiness and easy annoyance that is associated with adolescent depression^35^. Other ODD problems characterized as ‘headstrong’ (oppositional, disobedient), by contrast, lead to different outcomes. Furthermore, irritability mediates the association between foetal exposure to maternal depression and subsequent adolescent depression^29^. The potential importance of the demarcation is reflected in the DSM 5^36^ distinction within ODD between irritable and headstrong dimensions. While the different adolescent outcomes from irritability and headstrong symptoms are well established, we do not know whether they have different developmental origins. We hypothesised that the prenatal and postnatal exposures that we predict will be associated with depression from adolescence onwards, will be associated at age 7 years specifically with irritability and not with headstrong symptoms. In contrast to the irritability-headstrong specificity proposal, there have also been suggestions that many apparently specific psychiatric syndromes are simply a reflection of a broad dimension of general psychopathology, ‘p’. Therefore, in a second test of specificity we examined predictions to irritability after accounting for total emotional and behavioural problems, which provides an index of ‘p’ in young children.

Our hypotheses were: 1) elevated irritability will be predicted by a three-way interaction between prenatal and postnatal anxiety and maternal stroking, arising from adverse effects of mismatched prenatal and postnatal conditions in combination with low maternal stroking, 2) this effect will be seen after accounting for an index of general psychopathology, 3) this effect will not be seen with headstrong symptoms as the outcome, and 4) these associations will be seen in girls but not in boys.

## Method

### Study design and sample

The participants were members of the Wirral Child Health and Development Study (WCHADS), a prospective epidemiological longitudinal study starting in pregnancy with follow-up over several assessment points during infancy and childhood. This uses a two-stage stratified design in which a consecutive general population sample (the ‘extensive’ sample, N = 1233 recruited at 20 weeks gestation) is used to generate a smaller ‘intensive’ sample (n = 316) stratified by psychosocial risk, and both are followed in tandem. This enables intensive measurement to be employed efficiently with the stratified subsample, while weighting back to the extensive sample enables general population estimates to be derived. The analyses presented here were all conducted on the larger extensive sample which was identified from consecutive first-time mothers who booked for antenatal care at 12 weeks gestation between 12/02/2007 and 29/10/2008. The booking clinic was administered by the Wirral University Teaching Hospital which was the sole provider of universal prenatal care on the Wirral Peninsula, a geographical area bounded on three sides by water. Socioeconomic conditions on the Wirral range between the deprived inner city and affluent suburbs, but with very low numbers from ethnic minorities. The study was introduced to the women at 12 weeks of pregnancy by clinic midwives who asked for their agreement to be approached by study research midwives when they attended for ultrasound scanning at 20 weeks gestation.

### Ethical considerations

Ethical approval for the study was granted by the Cheshire North and West Research Ethics Committee on three occasions for longitudinal data collection, on the 27^th^ June 2006, reference number 05/Q1506/107, 7^th^ June 2010, reference number, 10/H1010/4, and on 22^nd^ December 2014, reference number, 14/NW/1484 and has therefore been performed in accordance with the ethical standards laid down in the 1964 Declaration of Helsinki and its later amendments. After obtaining written informed consent, the study midwives administered questionnaires, and informed consent was obtained again at later phases of the study.

Of those approached by study midwives, 68.4% gave consent and completed the measures, yielding an extensive sample of 1233 mothers with surviving singleton babies. Data were available on all 1233 from birth records and for anxiety measures at 20 weeks of pregnancy (‘20 weeks gestation’) and on 865 mothers for infant stroking at 9.3 (*SD* = 3.6) weeks (‘9 weeks’). Postnatal maternal anxiety was reported by 859 mothers at 9 weeks, by 708 at 14.3 (*SD* = 1.9) months (‘14 months’), and by 710 at 41.8 (*SD* = 2.3) months (‘3.5 years’). Child outcomes were available by maternal report on 667 children and by teacher report on 627 children at 88.2 (*SD* = 3.7) months (‘7 years’). The changes in sample sizes at each time point are attributable to sample attrition and missing data which were accounted for in all analyses.

### Measures

#### Maternal anxiety

Maternal anxiety was assessed at 20 weeks of pregnancy using the State Anxiety Scale^37^, a widely used maternal self-report measure which yields total scores ranging between 20–80. Postnatal maternal anxiety was assessed using the same measure at 9 weeks, 14 months and 3.5 years. Cronbach’s alphas across the four measurement points were between 0.79–0.86.

#### Maternal depression

Maternal symptoms of depression were assessed at 20 weeks gestation, and at 9 weeks, 14 months and 3.5 years using the Edinburgh Postnatal Depression Scale (EPDS) which yields scores from 0–30, and which has been used extensively to assess pre- and post-natal depression^38^. Cronbach’s alphas across the four measurement points were 0.77–0.90.

#### Maternal stroking

Maternal stroking was assessed by self-report using the Parent–Infant Caregiving Touch Scale (PICTS) at 9 weeks. The PICTS has 12 items covering a range of maternal touching and communication behaviours, that load on three factors of stroking, holding and affective communication^39^. Four stroking items assessed how often (1 = never, 2 = rarely, 3 = sometimes, 4 = often, 5 = a lot) mothers currently stroked their baby’s face, back, tummy, arms and legs. The stroking measure was administered to the intensive subsample at 5 weeks (n = 268), and to the extensive sample at 9 weeks (n = 838). Scores at 5 and 9 weeks were correlated r = 0.58, and the stroking factor showed substantial stability over the same period. In the current study, in which analyses were conducted only with the larger extensive sample, we used the 9-week maternal stroking measure. Cronbach’s alpha at age 9 weeks was 0.82. As reported previously we found no association between stroking at 9 weeks and observed maternal sensitivity at 29 weeks^26^ suggesting that the stroking measure assesses a distinct dimension from maternal responsivity to infant cues.

#### Child emotional and behavioural symptoms

Maternal reports of child symptoms were obtained at 7 years using the Child Behaviour Checklist (CBCL), which has been extensively employed in studies of child and adolescent emotional and behavioural disorders^40^. It has 99 items each scored 0 (not true), 1 (somewhat or sometimes true), and 2 (very true or often true). Teacher reports of child symptoms were obtained using the Teacher Report Form (TRF), also a widely used measure of child and adolescent symptomatology. Outcome variables were the highest of the maternal and teacher reports. If only one report from either the parent or the teacher was available, then that was used as the outcome variable. ODD dimensions of irritability and headstrong symptoms were generated based on the items previously identified by confirmatory factor analyses (CFA) in adolescents and adults^41,42^ and on CFA with data collected at ages 2.5, 3.5 and 5.0 years in this study^43^. Cronbach’s alpha for parent and teacher irritability scales were 0.73 and 0.78, and for headstrong scales they were 0.64 and 0.62. Items that comprise the Total Problem Score were used to generate the estimate of the general psychopathology ‘p’ factor. There is no established method for computing ‘p’. Using data from the Dunedin Multidisciplinary Health and Development Study, across 20 years from adolescence to midlife, Caspi *et al*. found that three higher-order factors (Internalising, Externalising and Thought Disorder) contributed to one General Psychopathology dimension which explained many apparently specific psychiatric disorders^44^. Thought disorder is not relevant during childhood, so we used the total problem score of the CBCL, which includes internalising and externalising, as a childhood index of ‘p’.

#### Confounders

Demographic and biological risks known to be associated with prenatal stressors and child mental health disorders were included as potential confounders. Variables generated at 20 weeks of pregnancy included mother’s age, her cohabiting/marital status, whether she had smoked during pregnancy, and whether or not she had stayed in full time education beyond age 18. Partner psychological abuse, which was the stratifier for the intensive sample, was assessed using a 20-item questionnaire covering humiliating, demeaning or threatening utterances in the partner relationship during pregnancy over the previous year^45^. The variable included in the models was defined as the highest of the partner to participant and participant to partner scores at 20 weeks pregnancy. Socioeconomic status was measured using the revised English Index of Multiple Deprivation (IMD)^46^; based on data collected from the UK Census in 2001.

### Statistical analyses

**Figure 1:**
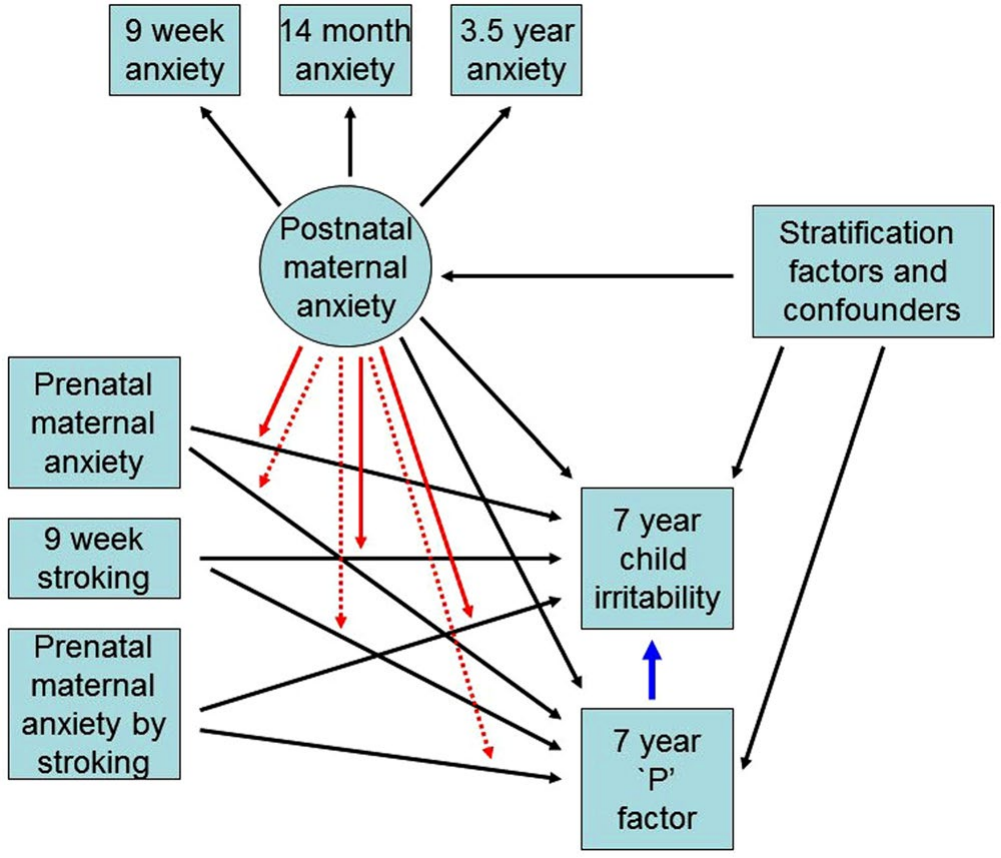
Summary of SEM for prediction of child irritability, accounting for general psychopathology, from prenatal and postnatal maternal anxiety and stroking. Main effects are shown in black, interactions in red and potential mediation of effects via general psychopathology ‘p’ in blue. Correlations among all observed predictor (exogeneous) variables are not shown.

To best estimate long term exposure to maternal anxiety during infancy we formed a factor from the 3 postnatal measurements of maternal anxiety collected from the full cohort. Figure 1 shows the form of a series of structural equation models (SEM) that were fitted in Stata 14 to examine irritability at age 7 years, each log-transformed to minimise skew (correlations among all predictor variables not shown). We used the program gllamm with 15-point adaptive quadrature for model fitting (www.gllamm.org)^47^, as this allowed us to fit all the effects shown in the Figure corresponding to main effects from risk factors, stratifiers and confounders (black), potential mediation of effects via the general psychopathology ‘p’ (blue) and interaction effects involving a latent variable (red) that are not easily fitted in other SEM programs. This capability comes at the cost of measures of goodness-of-fit.

We began by examining the estimated effects of 9-week maternal infant stroking as modifying the impact of the two-way interaction between maternal prenatal anxiety and the postnatal anxiety factor. The first model considered child irritability as the outcome. Estimated by using full maximum-likelihood, this model made use of data from 887 participants, who provided measures of postnatal anxiety and child psychopathology, including some with incomplete observations. The model also included the following potential confounders of the experience-behaviour relationship and predictors of participant attrition from the cohort: maternal age, smoking in pregnancy, education and marital status, and neighbourhood deprivation in addition to the stratifier, partner psychological abuse at 20 weeks pregnancy.

The second (shown in Fig. 1) and third more extended models included the prediction of the highest of the parent and teacher total CBCL score as an estimate of general psychopathology (‘p’), and its effects on irritability. These models required the total CBCL score to be included as a predictor, reducing the available sample size to 669 participants. Here, again, covariates for attrition were included.

The predicted effects of matched and mismatched prenatal and postnatal anxiety were examined using binary variables – high prenatal, low prenatal, high postnatal, low postnatal maternal anxiety - derived from median splits of the prenatal anxiety measure and the postnatal anxiety factor score. Matched groups were defined as either high prenatal-high postnatal or low prenatal-low postnatal anxiety, and mismatched groups as high prenatal-low postnatal or low prenatal-high postnatal maternal anxiety. The linear effects of maternal stroking in each of the 4 groups, and the pooled matched and mismatched groups were displayed with their corresponding regression Wald p–value for the interaction term.

## Results

Summary statistics for the measures included in the SEM are shown in Table 1. Not shown in the table, correlations between parent and teacher reports of child behaviours were irritability r = 0.23 (p < 0.001) and headstrong r = 0.31(p < 0.001). Table 2 shows the estimated effects from the SEM fitted to the whole sample accounting for the stratifier and confounders, whose effects are not shown, without the inclusion of the generic psychopathology factor ‘p’. There was a significant three-way interaction of pre- and postnatal anxiety and stroking on the prediction of child irritability. To confirm that the effects were specific to irritability, the total CBCL/TRF score was added to the model as a measure of ‘p’, as shown in Fig. 1. The results are shown in Table 3. As expected ‘p’ was strongly associated with irritability (p < 0.001) but the three-way interaction predicting ‘p’ was non-significant. Moreover, accounting for effects explainable by those on generic psychopathology reduced the magnitude of the pre- and postnatal anxiety by stroking interaction effect on irritability by just 12%, and with improved precision this interaction effect on irritability remained clearly significant (p = 0.003). The three-way interaction on the prediction of headstrong symptoms was by contrast entirely non-significant.

**Table 1:** Descriptive statistics for maternal and child variables.

	Boys			Girls		
	N	Mean	s.d.	N	Mean	s.d.
Prenatal anxiety	436	30.90	9.52	451	31.63	10.41
9 weeks postnatal anxiety	424	30.40	10.12	435	29.92	9.92
14 months postnatal anxiety	339	31.74	10.48	369	30.48	10.16
3.5 years postnatal anxiety	345	30.40	10.80	365	30.00	10.30
9 weeks stroking	436	3.88	0.70	451	3.86	0.70
CBCL Total – mother report	316	21.89	18.94	351	17.68	14.81
TRF Total – teacher report	296	16.63	19.06	331	9.31	13.18
Irritability – highest of mother/teacher	317	1.26	1.50	351	0.98	1.30
Headstrong- highest of mother/teacher	318	1.64	1.39	351	1.31	1.20
Stratum low	436	77%		451	75%	
Stratum mid		8%			7%	
Stratum high		16%			18%	
Maternal age <21 years	436	10%		451	12%	
Maternal age 21–30 years		56%			56%	
Maternal age >30 years		34%			32%	
Full time education only up to age 18	436	62%		451	67%	
Smoking – none	436	62%		451	64%	
Smoking before pregnancy		21%			19%	
Smoking during pregnancy		17%			18%	
No partner	436	17%		451	19%	
Most Deprived Quintile	436	37%		451	36%

**Table 2:** Summary of SEM for the interaction between prenatal-postnatal maternal anxiety and maternal stroking, predicting child irritability and headstrong symptoms at age 7 years.

	Irritability (N = 887)		Headstrong (N = 887)	
	Coefficient (95% CI)	p-value	Coefficient (95% CI)	p-value
Prenatal maternal anxiety 20 weeks gestation	0.038 (−0.050, 0.126)	0.397	0.004 (−0.091, 0.083)	0.925
Postnatal maternal anxiety factor	−0.149 −0.607, 0.309	0.523	0.151 (−0.157, 0.458)	0.336
Maternal stroking 9 weeks postnatal	−0.060 (−0.142, 0.022)	0.154	−0.048 (−0.130, 0.034)	0.252
Prenatal anxiety by stroking	−0.059 (−0.140, 0.022)	0.156	**−0.081** **(−0.159, −0.003)**	**0.043**
Postnatal anxiety by stroking	−0.067 (−0.375, 0.241)	0.668	0.001 (−0.322, 0.325)	0.994
Prenatal anxiety by postnatal anxiety	−0.129 (−0.410, 0.154)	0.372	−0.225 (−0.498, 0.047)	0.105
Prenatal anxiety by postnatal anxiety by stroking	**0**.**533** (**0**.**221**, **0**.**843**)	**0.001**	0.128 −0.169, 0.424	0.399

All analyses controlled for area deprivation, maternal age, marital status, partner psychological abuse and smoking during pregnancy. Child irritability and headstrong symptoms were from combined maternal and teacher report.

**Table 3:** Summary of SEM for the interaction between prenatal-postnatal maternal anxiety and maternal stroking, predicting child irritability and headstrong symptoms, including effects on, and of, general psychopathology estimated as total CBCL/TRF problem scores at age 7 years.

	Irritability (N = 669)		Headstrong (N = 669)		Total problem score (N = 669)	
	Coefficient 95% CI	p-value	Coefficient 95% CI	p-value	Coefficient 95% CI	p-value
Prenatal maternal anxiety 20 weeks	0.049 −0.022, 0.120	0.116	0.012 −0.060, 0.083	0.750	−0.034 (−0.120, 0.053)	0.443
Postnatal maternal anxiety factor	−0.171 −0.429, 0.086	0.179	−0.259 −0.536, 0.−018	0.067	**0.659** **(0.343, 0.975)**	**<0.001**
Maternal stroking 9 weeks postnatal	−0.041 −0.108, 0.025	0.288	−0.031 −0.097, 0.036	0.371	−0.017 (−0.098, 0.063)	0.669
Prenatal anxiety by stroking	−0.047 −0.112, 0.119	0.333	**−0.076** **−0.141, −0.011**	**0.021**	−0.010 (−0.088, 0.068)	0.801
Postnatal anxiety by stroking	−0.074 −0.193, 0.340	0.337	0.215 −0.068, 0.499	0.137	−0.311 (−0.658, 0.034)	0.077
Prenatal anxiety by postnatal anxiety	−0.131 −0.343, 0.087	0.367	**0.239** **−0.448, 0.010**	**0.041**	0.150 (−0.134, 0.434)	0.300
Prenatal anxiety by postnatal anxiety by stroking	**0.388** **0.145, 0.635**	**0.002**	−0.002 −0.238, 0.234	0.986	0.095 (−0.206, 0.397)	0.536
General psychopathology	**0.659** **0.343, 0.975**	**<0.001**	**0.701** **0.377, 10.026**	**<0.001**		

**Figure 2:**
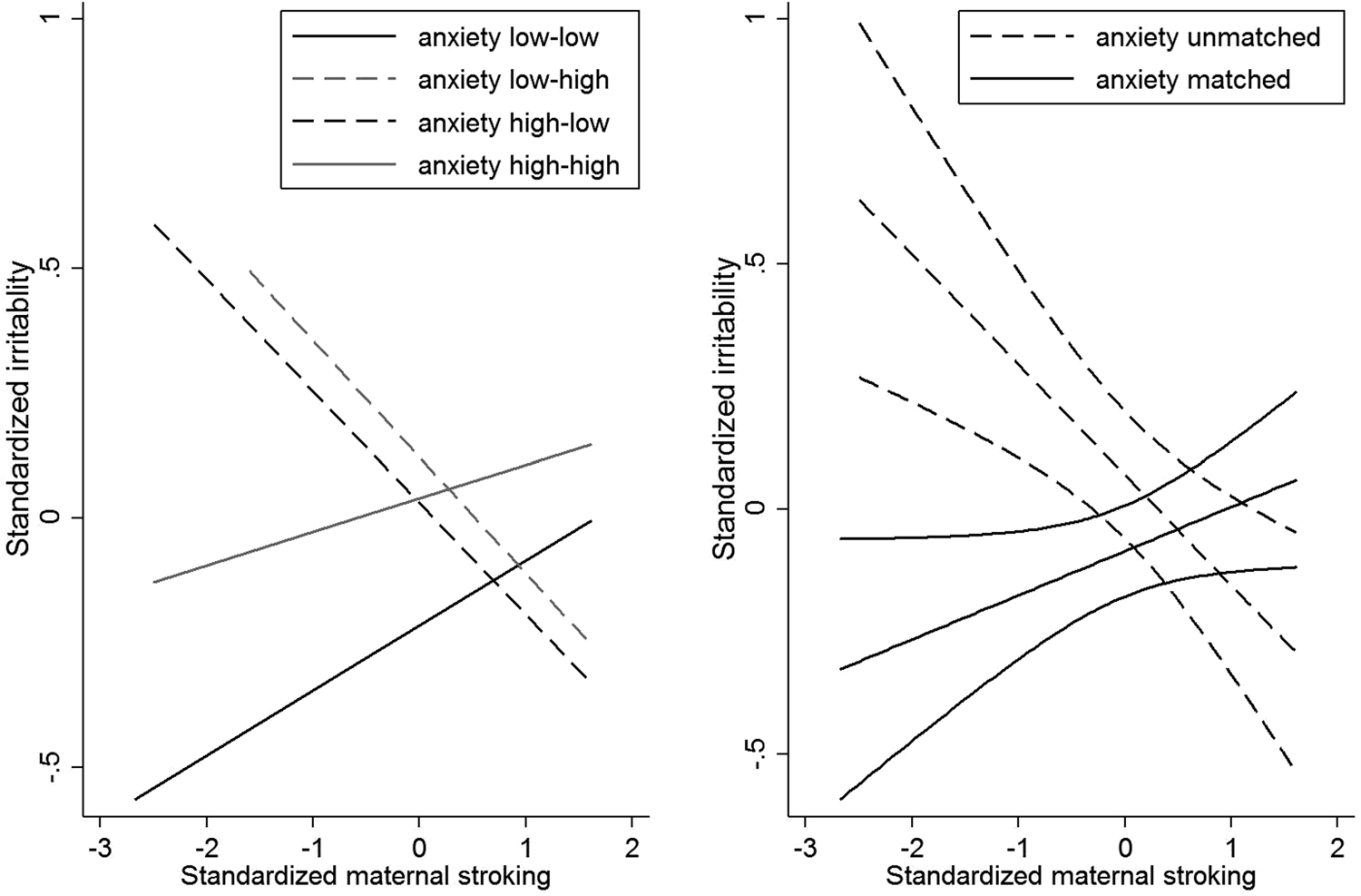
Associations between maternal stroking at 9 weeks and child irritability at 7 years, in matched and mismatched prenatal and postnatal maternal anxiety groups. In the left hand panel the dotted regression lines show the association between maternal stroking age 9 weeks and irritability at age 9 years in each mismatched maternal anxiety group (low prenatal - high postnatal; high prenatal - low postnatal) and the solid lines in each matched group (low prenatal - low postnatal; high prenatal - high postnatal). The groups were generated using medians of the maternal anxiety distributions. The right hand panel shows the regression lines and 95% confidence intervals in the combined mismatched and matched groups.

To examine the form of this interaction we tested the relationship of stroking to irritability in the four groups created by combining the binary high vs low variables for pre- and postnatal anxiety, among which two had matching pre and postnatal conditions and two were unmatched. Consistent with the strong continuity between prenatal and postnatal anxiety, the two matched groups (low – low, N = 294, high – high N = 307), were larger than the mismatched groups (low – high, N = 125, high – low, N = 161). The left-panel of Fig. 2 shows that in both mismatched groups there was a strong negative association between maternal stroking and child irritability, such that the highest irritability scores were predicted by a combination of maternal anxiety mismatches and low maternal stroking. In the presence of high maternal stroking, by contrast, irritability scores were lower in the mismatched groups than in infants exposed to persistent prenatal-postnatal maternal anxiety. There were some indications that stroking was associated with a modest increase in irritability symptoms in the matched groups. The right-hand panel shows the relationships when formed into just two groups of matched and unmatched environments which gave a highly significant simple interaction with maternal stroking (p < 0.001). By contrast with the findings for maternal anxiety, the three way interaction between prenatal depression, postnatal depression and maternal stroking predicting irritability was non-significant (coefficient = 0.187, 95% CI −0.059–0.433, p = 0.136).

Possible sex differences in the effects were examined by running the SEM models in girls and boys separately. Fitting the model to the sample of girls (n = 451) there was a larger 3-way interaction effect than in the whole sample (coefficient 0.372, p = 0.007 without p-factor; 0.309, p = 0.004 with p-factor included). Among the boys (n = 436), the interaction coefficient was substantially smaller and non-significant (coefficient 0.138, p = 0.508 without p-factor; 0.123, p = 0.490 with p-factor included). In separate linear regression models, for girls, maternal stroking explained 15.3% of the variance in irritability scores (p < 0.001) in the mismatched prenatal-postnatal anxiety groups, and there was a small effect in the opposite direction explaining 1.7% of the variance, in the matched groups (p = 0.041). In boys, by contrast, maternal stroking explained 0.2% of the variance in the mismatched groups (p = 0.639) and 0.0% of the variance in the matched groups (p = 0.558).

As all of the predictor variables were based on maternal reports, we looked for evidence that the associations we found were confined to maternal reports of child behaviours by examining separate models for mother and teacher reports. As we show in the Supplementary Materials, none of the models for the three-way interactions among scores, prenatal anxiety by postnatal anxiety by maternal stroking was significant. By contrast both models for the two-way interactions, match-mismatch binary variable by maternal stroking scores, were significant (mother report, regression Wald p = 0.035, teacher report regression Wald p = 0.019). As can be seen in the Supplementary Materials, the figures illustrating the interactions were very similar to each other, and to the figure for the combined scores shown in the paper.

## Discussion

We conducted analyses based on evolutionary hypotheses for foetal predictions of later environments (PAR hypothesis) and sex-biased parental investment (T-W hypothesis). We found that mismatched prenatal-postnatal maternal conditions, together with low levels of maternal tactile stimulation, were associated with high irritability, as predicted in hypothesis 1. Strikingly, as we show in the left hand panel of Fig. 2, the effect of low maternal stroking was seen to a similar degree irrespective of whether the pre-post mismatch was from low to high, or from high to low maternal anxiety. Furthermore, in the presence of low stroking, those with only prenatal or only postnatal exposure had higher irritability scores than those exposed to both. Hypotheses 2 and 3 made predictions based on specificity, that the reported associations did not merely reflect an effect on overall psychopathology, and that they were specific to the irritability, but not the headstrong, symptoms of oppositional defiant disorder. Both were supported. Although the predicted three-way interaction was found in analyses of the whole sample, in separate analyses of boys and girls, it was only present in girls. In view of the sample size we did not test the four-way interaction. As can be seen in Tables 2 and 3, there was not an overall match-mismatch effect of prenatal and postnatal maternal anxiety. Inspection of Fig. 2 suggests the reason for this, which is that at high levels of postnatal stroking the mismatch groups had the lower levels of irritability symptoms than the matched, and especially the high prenatal-high postnatal anxiety group.

It seems, therefore, that in addition to the many and complex social and cultural influences on the development of children’s behaviours, biological mechanisms seen across many species, and perhaps present from early in evolution, are also involved. These mechanisms probably reflect that Darwinian fitness entailed thriving in the context of both favourable and unfavourable environments. The result is an effect that runs counter to straightforward concepts of low and high-risk environments. The poorest outcomes in the girls, were not as would be predicted if risks were additive, in the presence of high prenatal anxiety followed by high postnatal anxiety. Rather, they were seen following mismatched combinations, either of high maternal anxiety followed by low anxiety, or *vice versa* of low followed by high, and in the absence of the protective effect of high maternal stroking. By contrast matched conditions, whether of the most favourable of low anxiety during pregnancy and after birth, or unfavourable of high anxiety during pregnancy and after birth, were associated with similar levels of irritability. Equally, while mismatched exposures conferred vulnerability in the presence of low stroking, there were indications that mismatched groups exposed to high maternal stroking had the best outcomes. This is consistent with the ‘differential susceptibility’ hypothesis which predicts not that individuals are more or less vulnerable to environments, but more or less affected by them, ‘for better’ in the presence of supportive environments, and ‘for worse’ in unfavourable environments. Recent animal evidence indicates that prenatal stress may have such ‘for better/for worse’ effects depending on exposure to tactile stimulation. Prairie voles exposed to high prenatal stress and cross-fostered to low contact postnatal rearing had the highest levels of anxiety-like behaviours while those who received high contact rearing had the lowest anxiety-like behaviours^48^. There was prenatal stress by rearing interaction, but no main effect of prenatal stress.

The finding that maternal mismatched anxiety in combination with low tactile stimulation predicted irritability only in girls, is consistent with an accumulating literature on sex-specific effects of early life stress. Findings from both the animal^14^ and human^21^ literature have shown that prenatal stress is associated with offspring anxiety and depression, an effect which is specific to females. Additionally, in rodent studies of prenatal stress, an adrenalectomy of the pregnant dams, which prevents glucocorticoid signaling, eliminated the effects of prenatal stress on female depression/anxiety behaviours^15^. This finding suggests that glucocorticoid mechanisms mediate the effects. Indeed, a recent human literature on effects of maternal prenatal cortisol has provided evidence for a glucocorticoid-mediated risk for adolescent depression in females. Prenatal cortisol has been associated with female adolescent depression and anxiety^19,21^, and also with early behavioural markers of depression, such as negative emotionality^17,18^ and a fearful temperament^19^. Here, we demonstrate a prediction to female irritability at age 7, an early marker of depression^34^ and mediator of associations between prenatal stress and adolescent depression^29^. Our findings extend current understanding of prenatal stress effects in females and suggest that the combination of low-prenatal high-postnatal anxiety, or *vice-versa*, creates risk for adolescent female depression in the context of low maternal stroking. It is possible that the sex-specificity of this association could contribute to the well-established sex-difference in adolescent affective disorders, where rates are much higher in females than males^49^.

The absence of a mismatch by stroking effect of maternal depressive symptoms adds to an inconclusive body of evidence on foetal programming. For example O’Connor *et al*., using data from the large ALSPAC study (N = 6,996) found that prenatal maternal anxiety, but not prenatal depression, predicted emotional and behavioural problems up to age 81 months^50^. On the other hand follow up of the ALSPAC sample to age 18 years (N = 4,303) revealed associations both of prenatal anxiety and prenatal depression with anxiety disorders^51^. In relation to prenatal mismatch effects we have reported that the low prenatal depression high postnatal depression mismatch predicts elevated NR3C1 methylation at age 14 months^52^. Whether there are distinctive outcomes associated with each of maternal anxiety and maternal depression remains to be established.

Strengths of the study include that the analyses were conducted on a large, general population, prospective sample, that they were based on repeated measurement of postnatal exposure to maternal anxiety, and that the outcome was assessed from both maternal and teacher reports. As we found in this study, parent-teacher agreement on child externalising behaviours is generally low, probably with many contributing factors including random measurement error, reporter bias, contextual differences and sex effects^53^. In the case of irritability, some children may have an intense emotional response to parental discipline leading to a high score on parental report, whereas other children have large responses to peer challenges in school, resulting in higher teacher reports. Combining parent and teacher reports is therefore very common in studies of externalising disorders, in order to maximise the capture of behaviours of interest across contexts^53,54^. Complex computational approaches for combining scores from different informants have been proposed, however these have not been shown to be superior to the simple method adopted in this study of using the score from whichever informant rated the higher^55^.

Limitations include that prenatal maternal anxiety was assessed on one occasion only, and that all the predictors, as well as some information on child outcomes, were by maternal report. While common method variance across predictor and outcome measures can inflate main effects, it is unlikely to give rise to moderator effects. Nevertheless we examined the question further in separate analyses using mother and teacher reports, shown in the Supplementary Materials. These were consistent in indicating that the effects identified were not an artefact of having an informant common across predictor and outcome. However, while the two way interactions yielded very similar significant findings to each other and to the analyses using combined ratings, analyses examining the three way interaction failed to demonstrate an effect based either on mother or on teacher reports, which was shown using combined reports. It is possible that the more highly skewed distributions generated from each informant, each with less information and thus power than the combined measure, made the models with coefficients for the product of 3 continuous variables less stable than the two way binary by continuous variable interaction.

Although we have shown that maternal stroking moderates associations between prenatal anxiety and depression and outcomes at 29 weeks, 14 months, 2.5 and 3.5 years, consistent with validity, we do not know that these self-reports are accurate. Agreement would add confidence that they are, although observational measures are limited by restricted coverage over place and time, and so cannot straightforwardly be considered as ‘gold standard’. As in the case of temperament research in infancy, in the absence of an agreed ‘gold standard’, self-report and observational measures perform complementary functions^56^ and further investigation of maternal stroking in infancy is likely to be approached similarly. A further limitation is that the main analyses showed interactions between maternal anxiety and stroking scores without providing evidence as to whether the interactive effects occurred across the distributions. The figures were based on widely used, but arbitrary, median thresholds, and so do not provide information on effects in relation to other thresholds. In particular, it cannot be inferred that the same mismatch effects would be observed in relation to higher thresholds or diagnosable anxiety disorders. A state anxiety measure was used at 20 weeks gestation to assess timing effects of state anxiety, however we did not include a measure of trait anxiety which would have enabled us to examine the role of state anxiety more precisely. Finally, our sample was not sufficiently powered to test a four-way interaction between maternal prenatal anxiety, postnatal anxiety, stroking and sex. Instead, we utilised three-way interactions and separate models for males and females. There were large differences between the three-way effects in males and females, and not just a statistical significance in one sex but not the other. However, without formally testing a four-way interaction we cannot interpret the findings as showing a sex difference.

The aim of this paper was to test behavioural hypotheses based on epigenetic and evolutionary considerations, however we are unable to draw firm conclusions regarding epigenetic or evolutionary effects based on our findings. Although the rationale for investigating maternal stroking in this study was the animal evidence for epigenetic effects of tactile stimulation on HPA axis regulation, in humans, tactile stimulation has many other effects. Characteristic patterns of prefrontal cortex and limbic activations have been shown in response to stroking with a pleasant stimulus, such as velvet, contrasted with a neutral or unpleasant stimulus, such as sandpaper^57^.

The implications of the findings need to be viewed in the context of continued debate concerning the applicability of the PAR hypothesis in humans. As Bateson *et al*. commented, “In mammals the cues to developing offspring are often provided by the mother’s plane of nutrition, her body composition or stress levels. This hypothetical effect in humans is thought to be important by some scientists and controversial by others”^6^. Further investigation, and in particular replication, are needed. With this caveat we outline four main implications of the findings. First, our findings suggest that variations in maternal emotional symptoms confer risk in a more complex way than previously thought because they contribute to, or reflect, processes that are adaptive or maladaptive depending on prior and later experiences. Second, early interventions may need to target not only mothers with elevated symptoms, but also those in pregnancy with few symptoms, but who may be at risk for onset postpartum, and also those whose prenatal symptoms improve following the birth of their child. Equally, these vulnerable groups may also be particularly responsive to early intervention. Third, in the context of available evidence for foetal origins of adolescent and hence adult depression, and for child irritability as a key mediator, the findings have the potential to further illuminate and provide the basis for childhood prevention of later depression. Finally, the study highlights how joint consideration of narrow and broad symptom profiles, within a longitudinal design, and testing mechanism-based hypotheses, can help clarify psychopathological phenotypes.

## Supplementary information

Supplementary Materials

## Data Availability

Information on how to apply for access to the data in line with the UK MRC policy on data sharing is provided at the study website, https://www.liverpool.ac.uk/psychology-health-and-society/research/first-steps/ by clicking on the ‘For Researchers’ button.
