# Supplementary material for: Predictions of children’s emotionality from evolutionary and epigenetic hypotheses
Source: Sci Rep. 2019 Feb 21;9:2519. doi: 10.1038/s41598-019-39513-7 (PMC6384926; doi:10.1038/s41598-019-39513-7)
Supplement: Supplementary file 1 — Supplementary Materials [file 41598_2019_39513_MOESM1_ESM.pdf]

Supplementary Materials – 'Predictions of children's emotionality from evolutionary and epigenetic hypotheses'

Jonathan Hill, Andrew Pickles, Nicola Wright, Elizabeth Braithwaite, Helen Sharp

As outlined in the main paper, the combining of reports, taking the highest of parent and teacher ratings of child behaviours, is widely used and has a sound evidential basis. In order to examine whether models based on mother report yielded stronger effects than those based on teacher report, and therefore may have arisen from comment method variance, we present here the single informant analyses.

As shown in Table S1 the available sample sizes for parent and teacher irritability assessments were slightly smaller than for the highest score combination. The mean score and thus also the information, given their skew, was substantially less for teachers than parents.

Table S1 Summary Statistics for Combined and Single Informant Outcomes

	Combined	Mother	Teacher
Irritability	N=770<br>M=1.16<br>SD=1.42	N=765<br>M=1.00<br>SD=1.35	N=724<br>M=0.37<br>SD=0.96
Headstrong	N=771<br>M=1.51<br>SD=1.33	N=763<br>M=1.35<br>SD=1.23	N=724<br>M=0.50<br>SD=1.11

We applied the same log transformation to the single informant reports as that used in the main paper for the combined report. The main paper presented two analyses. The first examined the 3-way interaction of the continuous scores of prenatal and postnatal stress and maternal stroking, including all the lower order interaction terms. While giving target interaction parameters in the same direction, these analyses gave non-significant estimates for both parent (p=.357) and teacher (p=.787) reports of child irritability.

The second analyses took advantage of mismatch theory and combined matched and mismatched stress quadrants, as defined by median scores of pre and post-natal stress, thereby reducing the complexity of the analysis and providing a simple two-way interaction as the target parameter. Figure 2 in the main paper illustrates the two way interaction for the combined reports of child irritability, in which low maternal stroking was associated with higher irritability specifically in the prenatal-postnatal anxiety mismatched groups. Applied to each informant, this approach yielded significant two-way interaction terms for both parent (p=.035) and teacher (p=.019) ratings of irritability. The figures illustrating these interactions shown in Figure S1 were very similar to each other, and to the main paper Figure 2 representing the interaction predicting combined mother and teacher scores.

These were consistent in indicating that the effects identified were not an artefact of having an informant common across predictor and outcome. However, in contrast to the two way interactions, analyses examining the three way interaction failed to demonstrate an effect based either on mother or on teacher reports, that was shown using combined reports. It is possible that the more highly skewed distributions generated from each informant, each with less information and thus power than the combined measure, made the models with coefficients for the product of 3 continuous variables less stable than the two way binary by continuous variable interaction.

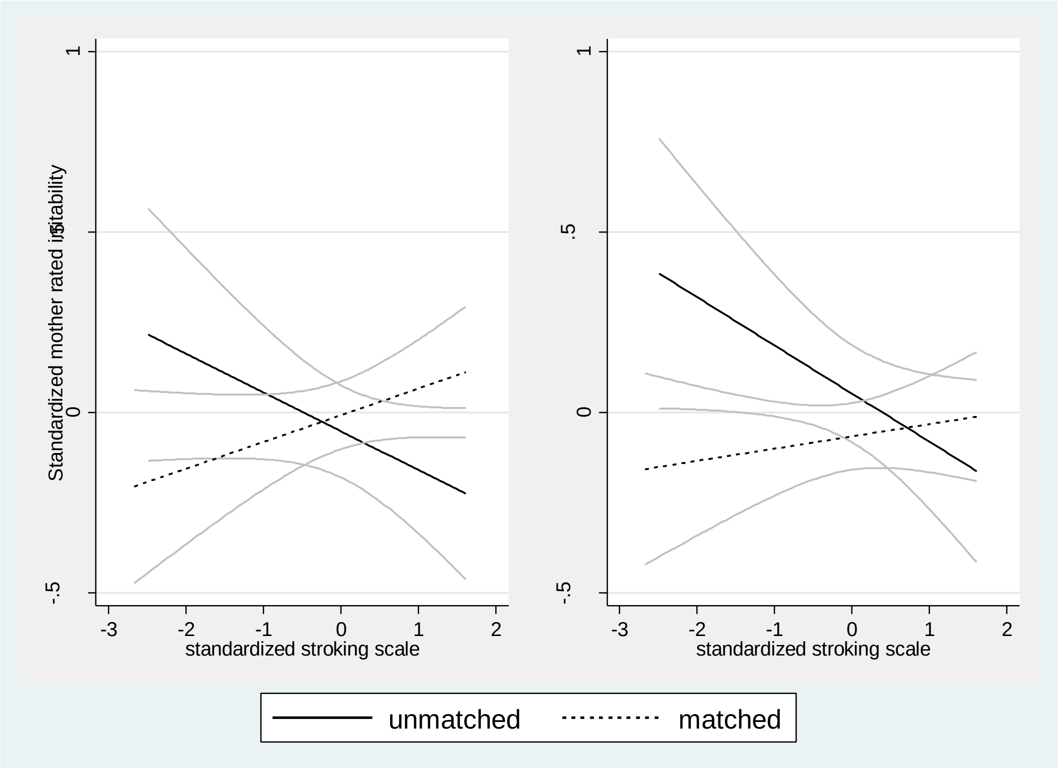

Figure S1 Associations between maternal stroking and child irritability, contrasting matched and mismatched prenatal-postnatal anxiety groups, based on teacher (left hand panel) and mother (right hand panel) reports.
